# A Population-Based and Clinical Cohort Validation of the Novel Consensus Definition of Metabolic Hyperferritinemia

**DOI:** 10.1210/clinem/dgad749

**Published:** 2023-12-20

**Authors:** Wen-Yue Liu, Li-You Lian, Huai Zhang, Sui-Dan Chen, Xin-Zhe Jin, Ni Zhang, Chen-Hui Ye, Wen-Ying Chen, George Goh Boon Bee, Fu-Di Wang, Luca Miele, Elena Corradini, Luca Valenti, Ming-Hua Zheng

**Affiliations:** Department of Endocrinology, the First Affiliated Hospital of Wenzhou Medical University, Wenzhou 325000, China; Wenzhou Key Laboratory of Diabetes Research, the First Affiliated Hospital of Wenzhou Medical University, Wenzhou 325000, China; MAFLD Research Center, Department of Hepatology, the First Affiliated Hospital of Wenzhou Medical University, Wenzhou 325000, China; Institute of Hepatology, Wenzhou Medical University, Wenzhou 325000, China; Key Laboratory of Diagnosis and Treatment for the Development of Chronic Liver Disease in Zhejiang Province, Wenzhou 325000, China; Biostatistics and Medical Quality Management Office, the First Affiliated Hospital of Wenzhou Medical University, Wenzhou 325000, China; Department of Pathology, the First Affiliated Hospital of Wenzhou Medical University, Wenzhou 325000, China; Department of Laboratory, the First Affiliated Hospital of Wenzhou Medical University, Wenzhou 325000, China; MAFLD Research Center, Department of Hepatology, the First Affiliated Hospital of Wenzhou Medical University, Wenzhou 325000, China; Institute of Hepatology, Wenzhou Medical University, Wenzhou 325000, China; Key Laboratory of Diagnosis and Treatment for the Development of Chronic Liver Disease in Zhejiang Province, Wenzhou 325000, China; MAFLD Research Center, Department of Hepatology, the First Affiliated Hospital of Wenzhou Medical University, Wenzhou 325000, China; Institute of Hepatology, Wenzhou Medical University, Wenzhou 325000, China; Key Laboratory of Diagnosis and Treatment for the Development of Chronic Liver Disease in Zhejiang Province, Wenzhou 325000, China; MAFLD Research Center, Department of Hepatology, the First Affiliated Hospital of Wenzhou Medical University, Wenzhou 325000, China; Institute of Hepatology, Wenzhou Medical University, Wenzhou 325000, China; Key Laboratory of Diagnosis and Treatment for the Development of Chronic Liver Disease in Zhejiang Province, Wenzhou 325000, China; Department of Gastroenterology and Hepatology, Singapore General Hospital, Singapore 169608, Singapore; The Fourth Affiliated Hospital, School of Public Health, Zhejiang University School of Medicine, Hangzhou 310000, China; The First Affiliated Hospital, Basic Medical Sciences, School of Public Health, Hengyang Medical School, University of South China, Hengyang 421001, China; Department of Internal Medicine Medical and Surgical Sciences, Fondazione Policlinico A. Gemelli IRCCS, Università Cattolica di Roma, Rome 00168, Italy; Department of Medical and Surgical Sciences, Università degli Studi di Modena e Reggio Emilia, Modena 41100, Italy; Internal Medicine and Centre for Hemochromatosis and Hereditary Liver Diseases, Azienda Ospedaliero-Universitaria di Modena-Policlinico, Modena 41100, Italy; Department of Pathophysiology and Transplantation, Università degli Studi di Milano, Milan 20121, Italy; Biological Resource Center and Precision Medicine Lab, Fondazione IRCCS Ca’ Granda Ospedale Maggiore Policlinico Milano, Milan 20121, Italy; Department of Transfusion Medicine, Fondazione IRCCS Ca’ Granda Ospedale Maggiore Policlinico Milano, Milan 20121, Italy; MAFLD Research Center, Department of Hepatology, the First Affiliated Hospital of Wenzhou Medical University, Wenzhou 325000, China; Institute of Hepatology, Wenzhou Medical University, Wenzhou 325000, China; Key Laboratory of Diagnosis and Treatment for the Development of Chronic Liver Disease in Zhejiang Province, Wenzhou 325000, China

**Keywords:** metabolic hyperferritinemia, metabolic dysfunction–associated fatty liver disease, metabolic syndrome, iron overload

## Abstract

**Context:**

There is limited data on the clinical significance of metabolic hyperferritinemia (MHF) based on the most recent consensus.

**Objective:**

We aimed to validate the clinical outcomes of MHF in the general population and patients with biopsy-proven metabolic dysfunction–associated fatty liver disease (MAFLD).

**Methods:**

The NHANES database and PERSONS cohort were included. MHF was defined as elevated serum ferritin with metabolic dysfunction (MD) and stratified into different grades according to ferritin (grade 1: 200 [females]/300 [males]—550 ng/mL; grade 2: 550-1000 ng/mL; grade 3: >1000 ng/mL). The clinical outcomes, including all-cause death, comorbidities, and liver histology, were compared between non-MHF and MHF in adjusted models.

**Results:**

In NHANES, compared with non-MHF with MD, MHF was related to higher risks of advanced fibrosis (*P* = .036), elevated albumin–creatinine ratio (UACR, *P* = .001), and sarcopenia (*P* = .013). Although the association between all grades of MHF and mortality was insignificant (*P* = .122), grades 2/3 was associated with increased mortality (*P* = .029). When comparing with non-MHF without MD, the harmful effects of MHF were more significant in mortality (*P* < .001), elevated UACR (*P* < .001), cardiovascular disease (*P* = .028), and sarcopenia (*P* < .001). In the PERSONS cohort, MHF was associated with more advanced grades of steatosis (*P* < .001), lobular inflammation (*P* < .001), advanced fibrosis (*P* = .017), and more severe hepatocellular iron deposition (*P* < .001).

**Conclusion:**

Both in the general population and in at-risk individuals with MAFLD, MHF was related with poorer clinical outcomes.

Ferritin, a protein responsible for iron storage, is the primary iron storage mechanism and is essential for iron homeostasis (1, 2). Ferritin makes iron accessible for essential cellular processes while shielding lipids, DNA, and proteins from iron's potentially deleterious effects (3, 4). In addition to the iron storage functions, previous studies have linked increased ferritin to the development of cancer and inflammation (5, 6). However, the leading cause of increased circulating ferritin levels in apparently healthy individuals is the presence of metabolic dysfunction (MD). In the general population, ferritin levels are associated with insulin resistance and the risk of developing type 2 diabetes mellitus (T2DM), and a faster progression of organ injury (such as hepatic and cardiovascular diseases) (7). In a study of 6497 patients, elevated ferritin was found to be significantly associated with the incidence of cardiovascular and cerebrovascular events and T2DM (8). In addition, a comprehensive study revealed that serum ferritin was closely related to the development of metabolic dysfunction–associated fatty liver disease (MAFLD), also known as nonalcoholic fatty liver disease (NAFLD) (9), and metabolic dysfunction–associated steatotic liver disease (10, 11). In this context, elevated serum ferritin and abnormal metabolism are intimately connected and should be treated as a unit.

Indeed, elevated serum ferritin levels could develop in individuals with MD in the presence of iron accumulation risk factors (9). Patients with hyperferritinemia combined with MD might be more susceptible to organ injuries. Although many previous studies have explored the connection between serum ferritin and metabolism (7, 12), systematic studies addressing this issue are needed.

Recently, a multidisciplinary expert panel has proposed specific diagnostic noninvasive criteria for the condition characterized by hyperferritinemia and MD, introducing the definition “metabolic hyperferritinemia” (MHF), and established a provisional grading of MHF severity (7). This consensus advanced clinical research on the epidemiology, genetics, pathophysiology, clinical relevance, and treatment of MHF by providing information on unmet needs, optimal design, clinically relevant outcomes, and by promoting collaborative studies on these topics, which would promote the clinical management of MHF. However, data on the prevalence of MHF and its impact on clinical outcomes are still lacking, as the new definition still needs real-world data validation.

Therefore, the present study aimed to validate the consensus definition and clinical significance of MHF in the general population from the National Health and Nutrition Examination Survey (NHANES) III and patients with biopsy-proven MAFLD in the Prospective Epidemic Research Specifically of NAFLD (PERSONS) cohort. Firstly, we compared the differences between MHF and non-MHF (with or without MD) regarding comorbidities and mortality in the NHANES III. Subsequently, we further explored the association between liver histological features and MHF in patients with MAFLD from the PERSONS cohort.

## Materials and Methods

### Study Cohorts

The NHANES is a national survey that provides extensive data on the nutrition and health of a US population outside of institutions. For this study, we analyzed publicly accessible NHANES III data from 1988 to 1994 (Fig. S1 (13)). The NHANES III was a national probability sample of 39 695 adults and children aged 2 months and older. It was conducted in 2 phases between 1988 and 1994 (14). The National Centre for Health Statistics has correlated data gathered from multiple National Centre for Health Statistics population surveys with death certificate records from the National Death Index. The public use–linked mortality files contain mortality follow-up data from the date of survey participation through December 31, 2019. The Ethical Review Board of the National Center for Health Statistics has endorsed the NHANES protocols, and all participants consented to use their information for research. The NHANES website (https://www.cdc.gov/nchs/nhanes/) provides additional details regarding the survey's design, methodology, and data.

Patients with biopsy-proven MALFD were recruited from the PERSONS project. Patients initially suspected of having MAFLD based on the presence of imaging-defined hepatic steatosis and/or persistently elevated serum transaminase levels with coexisting metabolic risk factors undergoing liver biopsy from 2016 to 2022 at the First Affiliated Hospital of Wenzhou Medical University were investigated. Only patients with biopsy-proven MAFLD were enrolled (Fig. S2 (13)). The diagnosis criteria were consistent with the consensus (7). This research was approved by the Institutional Ethics Committee of the First Affiliated Hospital of Wenzhou Medical University (no. 2016-0246) and was conducted in accordance with the Declaration of Helsinki. All enrolled patients provided written informed consent.

### Definitions

MHF was defined as serum ferritin concentrations >300 ng/mL in males and >200 ng/mL in females with MD, according to the recent consensus criteria (7). Further, the severity of MHF was graded based on circulating ferritin levels according to the consensus criteria (7). MHF grade 1: no significant iron accumulation (ferritin levels <550 ng/mL); MHF grade 2: mild iron accumulation (ferritin levels 550-1000 ng/mL); MHF grade 3: moderate or severe iron accumulation (ferritin levels >1000 ng/mL). MD was characterized by fatty liver, T2DM, and/or obesity, or 2 or more features of altered metabolism associated with insulin resistance (overweight or waist circumference ≥102/88 cm in men/women; blood pressure ≥130/85 mmHg or a certain medication regimen; plasma triglycerides ≥1.7 mmol/L; high-density lipoprotein-cholesterol <1.0/1.3 mmol/L in men/women; fasting levels of glucose >5.6 mmol/L; homeostatic model assessment of insulin resistance ≥2.7) (7). Individuals with other conditions that influence iron homeostasis were excluded (eg, haemochromatosis [transferrin saturation >50%], high alcohol intake [>60 g per day in men and >40 g per day in women within the past 6 months], end-stage chronic kidney disease [CKD, estimated glomerular filtration rate <15 mL/min/1.73 m^2^], advanced neoplasia, inflammatory disorders [active infections, active autoinflammatory disorders, C-reactive protein levels above the normal upper limit, advanced neoplasia, sepsis, and multiorgan failure], hyperferritinemia with unknown reasons and data missing). MAFLD was defined according to an international expert consensus statement (15). Patients were considered to have T2DM if they had a history of the condition or hypoglycemic drugs use or if their serum fasting plasma glucose (FBG) level was over 7.0 mmol/L or their glycated hemoglobin (HbA1c) level was over 6.5% or 2-hour oral glucose tolerance tests >11.1 mmol/L (16). Sarcopenia was defined as a skeletal muscle index greater than 1 SD below the respective sex-specific young adult (age 18-39 years old) means: 37.0% in men and 28.0% in women (17). Cardiovascular diseases (CVDs) in the NHANES III were defined by self-report by the participants. Body mass index (BMI) was determined by dividing weight (kg) by the square of the height (m^2^). Overweight was defined as a BMI ≥ 25 kg/m^2^, and obesity as a BMI ≥ 30 kg/m^2^ in the NHANES III. Insulin resistance was defined as the homeostatic model assessment of insulin resistance (calculated as FBG × fasting insulin/22.5) >2.7 (7, 18). The fibrosis-4 (FIB-4) index was used in the diagnostic protocol to evaluate advanced fibrosis (19). A FIB-4 value less than 1.30 was regarded as low risk for advanced fibrosis; a FIB-4 value greater than 2.67 was considered high risk for advanced fibrosis; and FIB-4 values between 1.30 and 2.67 were regarded as intermediate risk for advanced fibrosis (20). The clinical outcomes included all-cause death, comorbidities (liver fibrosis, elevated urine albumin–creatinine ratio [UACR], CVD, and sarcopenia) and liver histological features.

### Data Collection

In NHANES III, the following variables were regarded as covariates. Demographic variables such as age, sex, and ethnicity (Hispanic, non-Hispanic White, non-Hispanic Black, and other races) were extracted. Chronic comorbidities were also collected (such as hypertension, T2DM, and CVD). The complete blood count, serum albumin, alanine aminotransferase, aspartate aminotransferase, gamma-glutamyl transferase, estimated glomerular filtration rate, UACR, lipids, plasma glucose, HbA1c, creatinine, bilirubin, FBG, and uric acid were measured. Iron metabolism-related indexes such as serum ferritin, serum transferrin saturation, and serum total iron-binding capacity were enrolled. The NHANES website contains information regarding specimen collection, processing, quality assurance, and monitoring.

In the PERSONS cohort, the day before the liver biopsy, demographic information (age and sex), anthropometric data (height, weight, and waist circumference), and comorbidities (such as presence of T2DM and hypertension) were collected. On the day of the biopsy, peripheral venous blood samples were obtained after 8 hours of overnight fasting and then analyzed at the First Affiliated Hospital of Wenzhou Medical University. Venous blood samples included complete blood count and serum levels of ferritin, alanine aminotransferase, aspartate aminotransferase, gamma-glutamyl transferase, bilirubin, albumin, FBG, fasting insulin, lipids, creatinine, HbA1c, and uric acid.

### Evaluation of Liver Histological Feature

In the PERSONS cohort, liver biopsy guided by ultrasound was performed with a 16-gauge Hepafix instrument (Gallini). All biopsies were analyzed by experienced liver pathologists who were oblivious to the clinical and laboratory data of the participants. MAFLD lesions were scored according to the NAFLD activity score scoring system (21). The NAFLD activity score was determined as the sum of 3 histological components: steatosis (0-3), ballooning (0-2), and lobular inflammation (0-3). The stages of fibrosis were ranked between 0 and 4. Significant and advanced fibrosis were defined as fibrosis grade ≥2 and ≥3, respectively (22). The presence of iron in hepatocytes was evaluated by Perl's Prussian blue staining, and the severity of hepatocellular iron deposition was classified using a modified Scheuer system (23).

### Statistical Analysis

In NHANES III, the data were weighted according to the sample and multiperiod combination weights and adjusted for the variance underestimation caused by the design scheme. For continuous variables, data were expressed as weighted means with weighted standard errors or weighted median with weighted interquartile range, and for categorical variables, as real numbers with weighted proportions. Weighted logistical regression models adjusting for age, sex, and ethnicity were carried out to determine the relationship between MHF and comorbidities presented with an odds ratio (OR) and 95% CI. Weighted Cox regression models adjusting for age, sex, and ethnicity were performed to evaluate the association between MHF and mortality presented with a hazard ratio (HR) and 95% CI. Using weighted Kaplan–Meier curves, we compared the probability of survival outcomes between non-MHF and different MHF grades.

In the PERSONS cohort, continuous data were presented as the mean and SD if parametric or as the median and interquartile range if nonparametric. The categorical data were presented as number and percentage. Comparisons of continuous variables between groups were conducted with the Kruskal–Wallis test. Using the Chi-square test or Fisher's exact test, comparisons between frequencies or percentages were conducted. The relationship between MHF and liver histological features was analyzed using logistic regression adjusting for age and sex.

A 2-sided *P* < .05 was considered to be statistically significant. All analyses were conducted with R 4.2.1 (https://www.r-project.org/). The R package “jskm” (24) was used for weighted Kaplan–Meier analysis, and the R package “survey” (25) was used to analyze the complex survey samples in the NHANES III.

## Results

### Validation of MHF Outcomes in the NHANES III (From 1988 to 1994)

#### Clinical characteristics of the study population

A total of 12 779 individuals from the NHANES III were enrolled in our study. The baseline results of enrolled individuals from the NHANES III stratified by the grades of MHF are shown elsewhere (Table S1 (13)). We specifically classified non-MHF into 2 groups according to the presence or absence of MD, and compared them with MHF. There were 11 263 non-MHF (89.6%, including 1839 non-MD and 9424 MD individuals), 1256 grade 1 (9.0%), and 260 grade 2/3 (1.4%) MHF individuals. Males accounted for 47.1% in non-MHF and 57.7% in MHF. Individuals with MHF were also older (non-MHF: average age of 44.6 years old; MHF: average age of 55.2 years old; *P* < .001). In addition, the non-Hispanic Black population had a modest increase in MHF grade 2/3 (22.3%) compared with MHF grade 1 (13.5%).

#### Association between MHF and comorbidities

**Figure 1.**
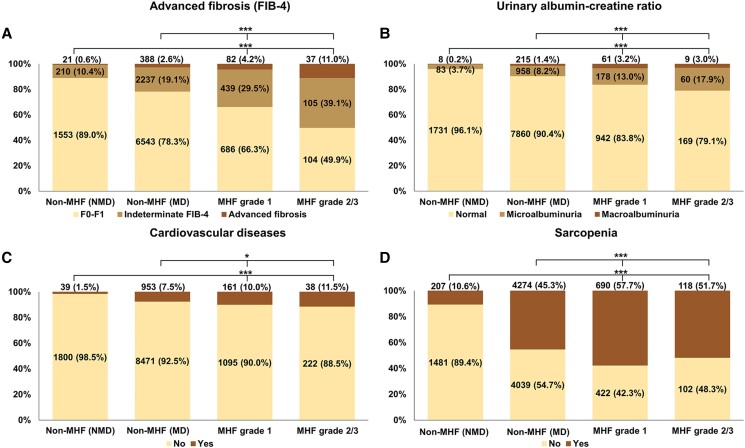
The clinical comorbidities according to the presence and severity of MHF in the NHANES III (from 1988 to 1994). The proportions of (A) advanced fibrosis, (B) urinary albumin–creatinine ratio, (C) cardiovascular disease, and (D) sarcopenia in MHF and non-MHF (with or without metabolic dysfunction). The data in the percentage column chart was real numbers (weighted proportion, %). *P < .05; **P < .005; ***P < .001. Abbreviations: MHF, metabolic hyperferritinaemia; MD, metabolic dysfunction; NMD, non-metabolic dysfunction; FIB-4, fibrosis-4.

We analyzed the association between MHF and comorbidities first (Fig. 1). Compared with non-MHF (with or without MD), the proportion of advanced fibrosis, elevated UACR, CVD, and sarcopenia was greater in MHF, and this proportion step increased according to MHF grades (all *P* < .05).

**Figure 2.**
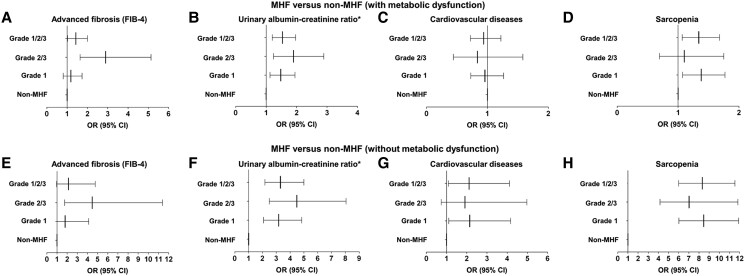
The association between clinical comorbidities and MHF in the NHANES III (from 1988 to 1994). The association between (A, E) advanced fibrosis, (B, F) urinary albumin–creatinine ratio, (C, G) cardiovascular disease, (D, H) sarcopenia and MHF compared with non-MHF (with or without metabolic dysfunction) in adjusted logistic regression models. The weighted logistic regression models adjusting for ethnicity (non-Hispanic white as a reference), age (continuous), and sex (female as a reference) were used to estimate the odds ratio (OR) and 95% CI. (A) Advanced liver fibrosis (OR grade 1 1.184, 95% CI 0.807-1.737, *P* = .378; OR grade 2/3 2.897, 95% CI 1.635-5.133, *P* = .001; OR grade 1/2/3 = 1.431, 95% CI 1.025-1.998, *P* = .036), (B) urinary albumin–creatinine ratio (OR grade 1 1.480, 95% CI 1.124-1.949, *P* = .006; OR grade 2/3, 1.898 95% CI 1.244-2.894, *P* = .004; OR grade 1/2/3 1.536, 95% CI 1.200-1.967, *P* = .001), (C) cardiovascular disease (OR grade 1 0.959, 95% CI 0.728-1.264, *P* = .761; OR grade 2/3 0.835, 95% CI 0.441-1.582, *P* = .573; OR grade 1/2/3 0.938, 95% CI 0.720-1.222, *P* = .629), (D) sarcopenia (OR grade 1 = 1.379, 95% CI 1.074-1.771, *P* = .013; OR grade 2/3 1.101, 95% CI 0.693-1.750, *P* = .677; OR grade 1/2/3 = 1.340, 95% CI 1.067-1.681, *P* = .013), (E) advanced liver fibrosis (OR grade 1 = 1.799, 95% CI 0.785-4.127, *P* = .161; OR grade 2/3 = 4.467, 95% CI 1.752-11.390, *P* = .002; OR grade 1/2/3 2.130, 95% CI 0.952-4.766, *P* = .065), (F) urinary albumin–creatinine ratio (OR grade 1 3.175, 95% CI 2.080-4.847, *P* < .001; OR grade 2/3 = 4.484, 95% CI 2.496-8.053, *P* < .001; OR grade 1/2/3 3.298, 95% CI 2.177-4.997, *P* < .001), (G) cardiovascular disease (OR grade 1 2.147, 95% CI 1.105-4.172, *P* = .025; OR grade 2/3 1.909, 95% CI 0.733-4.976, *P* = .180; OR grade 1/2/3 2.115, 1.087-4.114, *P* = .028), (H) sarcopenia (OR grade 1 8.465, 95% CI 6.022-11.900, *P* < .001; OR grade 2/3 = 7.020, 95% CI 4.166-11.830, *P* < .001; OR grade 1/2/3 8.323, 95% CI 5.999-11.550, *P* < .001). *We used urinary albumin–creatinine ratio normal to mildly increased (<30 mg/g) as reference, and calculated the influence of MHF grades in elevated urinary albumin–creatinine ratio (≥30 mg/g). Abbreviations: MHF, metabolic hyperferritinaemia; FIB-4, fibrosis-4.

We further explored the relationship between MHF and clinical comorbidities in the weighted logistic regression models adjusting for age, sex, and ethnicity (Fig. 2). Compared with non-MHF (with MD), MHF was related with higher risk of liver advanced fibrosis (OR 1.431, *P* = .036), elevated UACR (OR = 1.536, *P* = .001), and sarcopenia (OR 1.340, *P* = .013). However, the relationship between MHF and CVD was insignificant (OR 0.938, *P* = .629). When compared with non-MHF (without MD), MHF was associated with higher risks of elevated UACR (OR 3.298, *P* < .001), CVD (OR 2.115, *P* = .028), and sarcopenia (OR 8.323, *P* < .001). However, MHF was not related to a higher risk of advanced fibrosis (OR 2.130, *P* = .065).

We divided MHF into 2 groups according to the levels of serum ferritin (MHF grade 1 and grade 2/3) and compared them with non-MHF. When relative to non-MHF (with MD), different MHF subgroups had distinct effects on advanced liver fibrosis (OR grade 1 1.184, *P* = .378; OR grade 2/3 2.897, *P* = .001), elevated UACR (OR grade 1 1.480, *P* = .006; OR grade 2/3 1.898, *P* = .004), and sarcopenia (OR grade 1 1.379, *P* = .013; OR grade 2/3 1.101, *P* = .677). MHF was not related to CVD (OR grade 1 0.959, *P* = .761; OR grade 2/3 0.835, *P* = .573). When compared with non-MHF (without MD), the effects of the grades were also inconsistent in advanced liver fibrosis (OR grade 1 1.799, *P* = .161; OR grade 2/3 4.467, *P* = .002), elevated UACR (OR grade 1 3.175, *P* < .001; OR grade 2/3 4.484, *P* < .001) and sarcopenia (OR grade 1 8.465, *P* < .001; OR grade 2/3 7.020, *P* < .001). The relationship between MHF and CVD was weak (OR grade 1 2.147, *P* = .025; OR grade 2/3 1.909, *P* = .180).

#### Association between MHF and mortality

**Figure 3.**
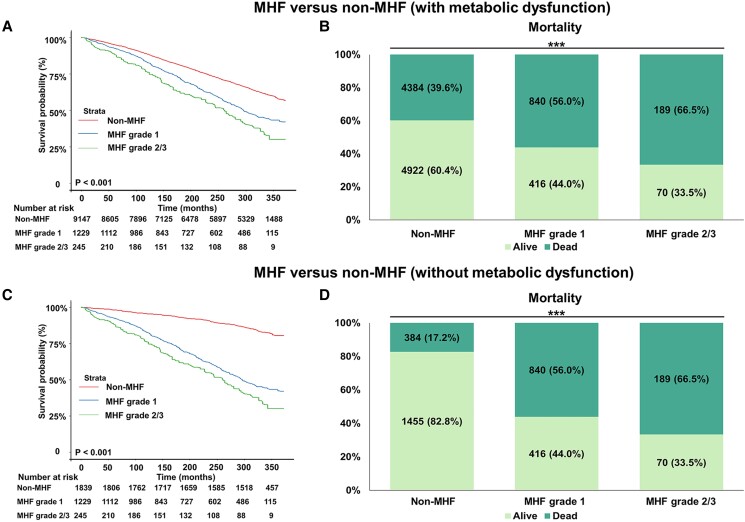
The weighted survival analysis of MHF and non-MHF (with or without metabolic dysfunction) in the NHANES III (from 1988 to 1994). The Kaplan–Meier curves of MHF and non-MHF (A, C) and the proportion of mortality in MHF and non-MHF (B, D). The data in the percentage column chart are real numbers (weighted percentage, %). *P < .05; **P < .005; ***P < .001. Abbreviations: MHF, metabolic hyperferritinaemia.

All patients were monitored for all-cause mortality during the follow-up till December 31, 2019. The weighted survival analysis showed MHF groups had higher mortality than non-MHF (with or without MD). The mortality in non-MHF with or without MD was 39.6% or 17.2%, in MHF grade 1 was 56.0% and in grade 2/3 was 66.5% (Fig. 3).

After adjusting for age, sex, and ethnicity, compared with non-MHF with MD, all grades of MHF were not related to higher mortality (HR 1.083, *P* = .122), see in Table 1. While stratified into different grades, grade 2/3 of MHF showed a significant increased risk of mortality (HR grade 1 1.054, *P* = .374; HR grade 2/3 1.279, *P* = .029). Compared with non-MHF without MD, MHF had a strong association with higher mortality (HR grade 1 1.400, *P* < .001; HR grade 2/3 1.686, *P* = .001; HR grade 1/2/3 1.432, *P* < .001). Furthermore, the incremental effects of MHF from grade 1 to grade 2/3 in mortality was proved by trend tests (*P* trend < .05).

**Table 1.** The association between MHF and mortality in adjusted Cox regression models in the NHANES III (from 1988 to 1994)

MHF grade	MHF vs non-MHF (with metabolic dysfunction)			MHF vs non-MHF (without metabolic dysfunction)		
	HR (95% CI)	*P*	*P* for trend	HR (95% CI)	*P*	*P* for trend
Non-MHF	Reference			Reference		
Grade 1	1.054 (0.939-1.184)	.374	.034	1.400 (1.204-1.628)	<.001	<.001
Grade 2/3	1.279 (1.026-1.594)	.029		1.686 (1.252-2.271)	.001	
Grade 1/2/3	1.083 (0.979-1.199)	.122		1.432 (1.230-1.666)	<.001	

We used the non-MHF as reference and calculated the weighted HR and weighted 95% CI for mortality in each MHF grade using weighted Cox regression models adjusting for ethnicity (non-Hispanic white as a reference), age (continuous) and sex (female as a reference).

Abbreviations: MHF, metabolic hyperferritinemia; HR, hazard ratio; NHANES, National Health and Nutrition Examination Survey.

### Validation of MHF Outcomes in Biopsy-Proven MAFLD Cohort

#### Clinical characteristics of study population

A total of 864 MAFLD patients confirmed by liver biopsy from 2016 to 2022 at the First Affiliated Hospital of Wenzhou Medical University were included, of which 414 (47.9%) were patients without MHF, 310 (35.9%) were patients with MHF grade 1, and 140 (16.2%) were patients with MHF grade 2/3 (Table S2) (13). MHF had a greater proportion of males than non-MHF (non-MHF: 63.0%; MHF: 81.3%; *P* < .001). There was no discernible difference in the age distribution of MHF grades (non-MHF: average age of 42.1 years old; MHF: average age of 42.3 years old; *P* = .755).

#### Association between MHF and liver histological features

**Figure 4.**
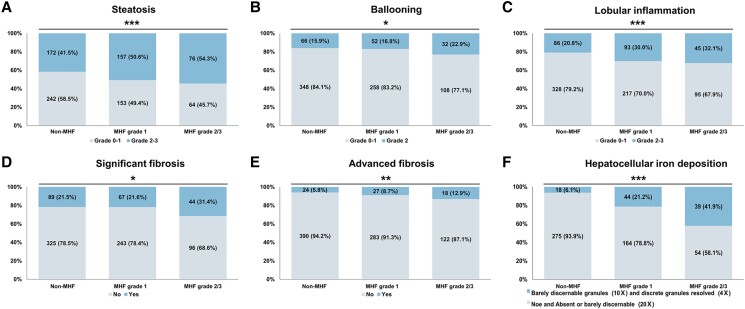
The proportion of liver histological features in patients with MAFLD with or without MHF in the PERSONS cohort. The proportion of (A) steatosis, (B) ballooning, (C) lobular inflammation, (D) significant fibrosis, (E) advanced fibrosis, and (F) hepatocellular iron deposition in MHF and non-MHF. The data in the percentage column chart are the numbers (percentage, %). The *P* values are *P* for trend: *P trend < .05; **P trend < .005; ***P trend < .001. Abbreviations: MHF, metabolic hyperferritinaemia; MAFLD, metabolic dysfunction-associated fatty liver disease.

**Figure 5.**
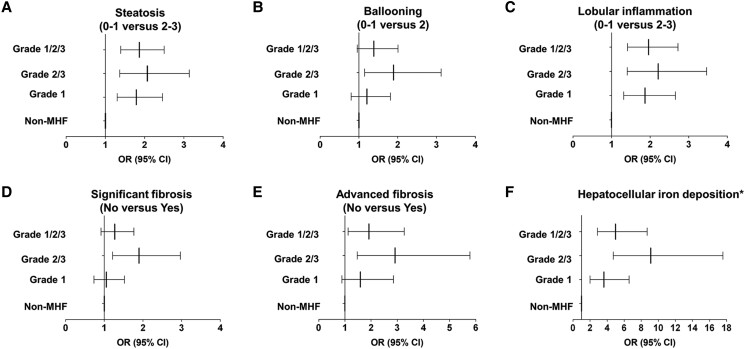
The association of liver histological features and MHF in patients with MAFLD in the PERSONS cohort. The association between (A) steatosis, (B) ballooning, (C) lobular inflammation, (D) significant fibrosis, (E) advanced fibrosis, (F) hepatocellular iron deposition, and MHF compared with non-MHF. The logistic regression models adjusting for age (continuous) and sex (female as a reference) were used to estimate the odds ratio (OR) and 95% CI. (A) steatosis (OR grade 1 1.787, 95% CI 1.301-2.454, *P* < .001; OR grade 2/3 2.068, 95% CI 1.363-3.138, *P* = .001; OR grade 1/2/3 1.865, 95% CI 1.392-2.499, *P* < .001), (B) ballooning (OR grade 1 1.205, 95% CI 0.800-1.814, *P* = .372; OR grade 2/3 1.891, 95% CI 1.146-3.120, *P* = .013; OR grade 1/2/3 1.385, 95% CI 0.956-2.006, *P* = .085), (C) lobular inflammation (OR grade 1 1.868, 95% CI 1.314-2.656, *P* = .001; OR grade 2/3 = 2.208 1.407-3.463, *P* = .001; OR grade 1/2/3 1.960, 95% CI 1.413-2.719, *P* < .001), (D) significant fibrosis (OR grade 1 1.056, 95% CI 0.733-1.522, *P* = .768; OR grade 2/3 1.900, CI 1.216-2.970, *P* = .005; OR grade 1/2/3 1.270, 95% CI 0.914-1.764, *P* = .154), (E) advanced fibrosis (OR grade 1 1.592, 95% CI 0.888-2.855, *P* = .118; OR grade 2/3 2.914, 95% CI 1.471-5.774, *P* = .002; OR grade 1/2/3 1.917, 95% CI 1.126-3.264], *P* = .017), (F) hepatocellular iron deposition (OR grade 1 3.626 , 95% CI 1.999-6.579, *P* < .001; OR grade 2/3 9.109, 95% CI 4.723-17.568, *P* < .001; OR grade 1/2/3 4.985, 95% CI 2.862-8.682, *P* < .001). *None and absent or barely discernable (20×) vs barely discernable granules (10×) and discrete granules resolved (4×). Abbreviations: MAFLD, metabolic dysfunction-associated fatty liver disease; MHF, metabolic hyperferritinaemia.

We compared the liver histological features between MHF and non-MHF in patients with MAFLD. Compared to patients without MHF, patients with MHF had more advanced grades of steatosis, ballooning, lobular inflammation, liver fibrosis, and hepatocellular iron deposition (*P* < .05, Fig. 4). We further explored the relationship between MHF and liver histological features in patients with MAFLD in the logistic regression model adjusting for age and sex (Fig. 5). Compared with non-MHF, MHF was associated with advanced grades of steatosis (OR 1.865, *P* < .001), lobular inflammation (OR 1.960, *P* < .001), advanced fibrosis (OR 1.917, *P* = .017), and more severe hepatocellular iron deposition (OR 4.985, *P* < .001). However, the relationship between MHF and ballooning (OR 1.385, *P* = .085) or significant fibrosis (OR 1.270, *P* = .154) was insignificant.

We further calculated the influences of different grades of MHF on liver histological features (Fig. 5). Different subgroups of MHF had distinct effects on steatosis (OR grade 1 1.787, *P* < .001; OR grade 2/3 2.068, *P* = .001), ballooning (OR grade 1 1.205, *P* = .372; OR grade 2/3 1.891, *P* = .013), lobular inflammation (OR grade 1 1.868, *P* = .001; OR grade 2/3 2.208, *P* = .001), significant fibrosis (OR grade 1 1.056, *P* = .768; OR grade 2/3 1.900, *P* = .005), advanced fibrosis (OR grade 1 1.592, *P* = .118; OR grade 2/3 2.914, *P* = .002), and hepatocellular iron deposition (OR grade 1 3.626, *P* < .001; OR grade 2/3 9.109, *P* < .001).

To clarify the impact of chronic viral hepatitis in MHF outcomes, we performed a sensitivity analysis in which 116 (13.4%) patients with MAFLD combined with chronicle viral hepatitis (HBV or HCV) were excluded (Fig. S3A) (13). These patients accounted for 15.0%, 11.9%, and 12.1% in non-MHF, and MHF grade 1 and 2/3, respectively (*P* trend > .05) (Fig. S3B (13)). In adjusted logistic regression models, there is no differences in the association between MHF and liver histological features before and after excluding these patients (Fig. S4 (13)).

## Discussion

In this study, we used the novel definition of MHF and validated its related clinical outcomes both in the general population from US based on NHANES III database and in the biopsy-proven MAFLD cohort from China. MHF accounted for 10.5% in the NHANES III and 52.1% in MAFLD biopsy cohort. MHF had a higher proportion of males than non-MHF in both cohorts. There were racial differences among MHF and non-MHF populations in the general US population. In NHANES III, compared with non-MHF (with or without MD), MHF was associated with higher risks for all-cause death, liver fibrosis, abnormal UACR, and sarcopenia. In the biopsy-proven Chinese MAFLD cohort, we found that MHF was associated with more severe liver histological damages, such as steatosis, lobular inflammation, fibrosis, and increased hepatocellular iron deposition. Our results suggested that both in the general population and at-risk individuals with MAFLD, MHF was related to poorer clinical outcomes, and the risk increased with grades.

Multiple studies had established that increased levels of serum ferritin could serve as a predictive indicator for metabolic syndrome (26) and T2DM (27). Excess of iron induced oxidative stress and damaged beta cells, resulting in impaired insulin secretion (28). Similarly, heightened ferritin levels could potentially indicate systemic inflammation with increased iron stores (29), increasing the risk of developing metabolic syndrome or aberrant glucose metabolism. This highlighted the close relationship between iron metabolism and metabolic abnormalities. Our study demonstrated the importance of treating the metabolic abnormalities and elevated serum ferritin as a whole in clinical practice.

In the present study, the non-MHF individuals from NHANES III were separated into with MD and without MD groups at the beginning of the study. We compared both groups with MHF in subsequent research to determine whether the poorer clinical outcomes were simply due to MD or MD-induced hyperferritinemia. Our study showed that patients with MHF had a greater mortality than non-MHF individuals (with or without MD), demonstrating that the risk stemmed not only from metabolic abnormalities but had a close relationship with alterations in iron metabolism. To better comprehend the reasons of MHF contributed to the increased risk of mortality, we further analyzed the relationship between MHF and clinical comorbidities.

We used both noninvasive indicators and liver pathological grades to evaluate liver damage and found that MHF was associated with more severe liver damage. Other studies have also found an association between elevated serum ferritin and hepatic morphological alterations (30-33). The findings above indicated that individuals with abnormal iron metabolism were more likely to experience abnormal liver pathology. However, these studies did not analyze the additional mechanism of liver pathological injuries caused by elevated ferritin from a metabolic standpoint. Our research, on the basis of previous studies, further emphasized the significance of metabolic factors in hyperferritinemia. The potential mechanism between MHF and clinical outcomes is still unclear. However, iron deposition mediated ferroptosis and lipotoxicity might play an important role in this pathological process (34). Researchers have found that excess fatty acids have been linked to a diminished ability of hepcidin to limit intestinal iron absorption while simultaneously increasing hepatic iron assimilation and tissue deposition, resulting in a subtle alteration in iron fluxes (35, 36). The relationship between MHF and tissue iron accumulation (such as liver) still needs further exploration. According to our results, higher grades of MHF (such as MHF grade 2/3) might be related to a higher risk of tissue iron accumulation. Due to the lack of magnetic resonance imaging data for hepatic iron accumulation, we could not infer whether MHF could be a noninvasive method for detecting tissue iron accumulation.

Additionally, we found that MHF was associated with the increment of UACR, which indicated that CKD might be a clinical manifestation of metabolic abnormalities and there was a close relationship between CKD and iron metabolism. Previous studies had also found the prognostic value of hyperferritinemia in the CKD population (37-39). Similarly, the most recent published expert consensus on MAFLD and CKD provides a framework for the early prevention and management of these 2 prevalent and interrelated diseases (40), further connecting metabolism and CKD. Iron overload in kidney would cause ferroptosis, a form of regulated cell death that is distinguished from other forms of cell death by the accumulation of iron, lipid peroxidation, and dense mitochondrial membranes (41). We discovered that patients with a higher grade of MHF had a greater proportion of aberrant UACR, possibly indicating specific renal pathology and the possible need for invasive examination. Renal biopsy is still the gold standard for assessing renal iron deposition and renal pathology. However, comparatively few clinical studies evaluated the relationship between renal iron deposition and renal pathological damage using renal biopsy. In the future, we need to investigate the effect of MHF on kidney injury in greater depth and then identify patients who are candidates for renal biopsy.

According to animal and human studies conducted previously, ferritin is closely linked to CVD (42-44). There was evidence that serum ferritin levels were associated with oxidized low-density lipoprotein, which is a risk factor for coronary heart disease (45, 46). In addition, important clinical correlations between MAFLD and the risk of CVD were identified by the expert consensus (47), which summarized the strong relationship between metabolism and CVD. However, our investigation showed a weak correlation between MHF and CVD. Although we did not identify a correlation between MHF and CVD, we remained skeptical of this result. The method of obtaining CVD diagnosis in the NHANES database might contribute to the weak results to some extent. The CVD data in the NHANES database was collected through questionnaires rather than clinical diagnosis, which might account for a particular bias. We could further select additional cardiovascular-related indicators, such as myocardial enzyme spectrum, electrocardiogram, coronary angiography, etc., to explore the correlation between CVD and MHF in greater depth. Taken together, we still thought the necessity of the close attention between iron overload and CVD. Individuals diagnosed with high level of MHF grade (eg, MHF grade 3) necessitated more strict monitoring of electrocardiograms, myocardial enzyme spectrum, echocardiography and coronary angiography and etc.

In addition to the above comorbidities, we also explored the relationship between MHF and muscle mass. We found a more significant proportion of patients with sarcopenia in MHF than in non-MHF, and this difference was more pronounced in the compassion between MHF and non-MHF without MD. Similar results were obtained in previous investigations (48, 49). As such, serum ferritin levels might be a predictor of sarcopenia and an essential link between sarcopenia and metabolic disorders. In addition, this association was affected by sex, age, and ethnicity. Exercise plus nutrition remain the main treatment for sarcopenia (50). MHF may become a novel screening method for high-risk groups of sarcopenia in the future, allowing for early diagnosis and early interventional treatment of this portion of the population. Further research is needed to determine whether sarcopenia combined with MHF requires additional iron deficiency therapy.

Our study demonstrated the strong association between iron metabolism and multiorgan diseases. Furthermore, previous research demonstrated there were interrelations between liver fibrosis, elevated UACR and sarcopenia (51-53). Among patients with MAFLD, elevated liver stiffness was associated with an increased risk of kidney complications (51). Sarcopenia was also linked to an increase in UACR (52). Does iron metabolism play a central role in the interrelations of these aforementioned diseases? This will be an interesting and important topic and further prospective and mechanism studies are needed.

The cornerstones of the clinical treatment of MHF are promoting a healthy lifestyle, a balanced diet, eating less processed foods, regular exercise, and consuming less alcohol. Separately, iron depletion therapy is one of the most common methods reducing the serum ferritin. Iron depletion by phlebotomy enhances insulin sensitivity in patients with NAFLD and hyperferritinemia, according to a case–control study (54). A randomized controlled study also confirmed that phlebotomy could reduce serum ferritin levels (55). Nevertheless, only patients with confirmed iron overload should be considered for iron depletion. Thus, when treating patients with MHF, determining whether the elevated serum ferritin level represented genuine iron overload remains one of the most challenging tasks. In the absence of genuine iron overload, the role of phlebotomy remains debatable.

This study has limitations that must be considered, such as the retrospective and cross-sectional nature. Future prospective study may be required to further validate our findings. The data in the NHANES III would not be updated any more. We chose NHANES III due to availability of liver ultrasound data. In the continuous NHANES, the diagnosis of fatty liver was based on fatty liver index. The absence of MRI data for the diagnosis of MHF was also one of the limitations of our study. As the number of MHF grade 3 patients was small, grade 2 was combined with grade 3 for analysis. Consequently, this study was unable to assess the effect of individual grade. To further investigate the clinical significance of MHF, a larger number of grade 3 individuals will be needed. We roughly analyzed whether the presence or absence of chronic viral hepatitis would affect the relationship between MHF and liver histological features. The findings revealed there was no bearing on our conclusions. However, our findings may be impacted by the small population of chronic viral hepatitis.

### Conclusion

In conclusion, we found that in a US general population and in Chinese patients with MAFLD, the presence of MHF is associated with more clinical comorbidities, higher risk of mortality and severe liver damages. Future studies should specifically examine whether incorporation of MHF evaluation in clinical practice may be useful to improve clinical outcomes by refining risk stratification and refining treatment strategies, which may include iron depletion in the most severe MHF grade associated with excess body iron. Multicenter and prospective studies are also warranted to validate the present results and identify the specific genetic and environmental risk factors for MHF.

## Data Availability

Some or all datasets generated during and/or analyzed during the current study are not publicly available but are available from the corresponding author on reasonable request.

## Abbreviations

BMI: body mass index
CKD: chronic kidney disease
CVD: cardiovascular disease
FBG: fasting plasma glucose
FIB-4: fibrosis-4
HbA1c: glycated hemoglobin
MAFLD: metabolic dysfunction–associated fatty liver disease
MD: metabolic dysfunction
MHF: metabolic hyperferritinemia
NAFLD: nonalcoholic fatty liver disease
NHANES: National Health and Nutrition Examination Survey
T2DM: type 2 diabetes mellitus
UACR: urine albumincreatinine ratio
